# Vasopressin Loading for Refractory Septic Shock: A Preliminary Analysis of a Case Series

**DOI:** 10.3389/fmed.2021.644195

**Published:** 2021-05-04

**Authors:** Kensuke Nakamura, Hidehiko Nakano, Hiromu Naraba, Masaki Mochizuki, Yuji Takahashi, Tomohiro Sonoo, Hideki Hashimoto, Toshikazu Abe, Mineji Hayakawa, Kazuma Yamakawa

**Affiliations:** ^1^Department of Emergency and Critical Care Medicine, Hitachi General Hospital, Ibaraki, Japan; ^2^TXP Medical Co., Ltd., Tokyo, Japan; ^3^Department of Emergency and Critical Care Medicine, Tsukuba Memorial Hospital, Tsukuba, Japan; ^4^Department of Health Services Research, Faculty of Medicine, University of Tsukuba, Tsukuba, Japan; ^5^Department of Emergency Medicine, Hokkaido University Hospital, Sapporo-shi, Japan; ^6^Department of Emergency Medicine, Osaka Medical College Hospital, Takatsuki, Japan

**Keywords:** critical care, septic shock, sepsis, vasopressin, loading

## Abstract

**Background:** Vasopressin is one of the strong vasopressor agents associated with ischemic events. Responses to the administration of vasopressin differ among patients with septic shock. Although the administration of a high dose of vasopressin needs to be avoided, the effects of bolus loading have not yet been examined. Since the half-life of vasopressin is longer than that of catecholamines, we hypothesized that vasopressin loading may be effective for predicting responses to its continuous administration.

**Methods:** We retrospectively analyzed consecutive cases of septic shock for which vasopressin was introduced with loading under noradrenaline at >0.2 μg/kg/min during the study period. Vasopressin was administered in a 1 U bolus followed by its continuous administration at 1 U/h. The proportion of patients with a negative catecholamine index (CAI) change 6 h after the introduction of vasopressin was set as the primary outcome. We defined non-responders for exploration as those with a mean arterial pressure change <18 mmHg 1 min after vasopressin loading, among whom none had a change in CAI <0.

**Results:** Twenty-one consecutive cases were examined in the present study, and included 14 responders and 7 non-responders. The primary outcome accounted for 71.4% of responders and 0% of non-responders, with a significant difference (*p* = 0.0039). Median CAI changes 2, 4, and 6 h after the administration of vasopressin were 0, −5, and −10 in responders and +20, +10, and +10 in non-responders, respectively. CAI was not reduced in any non-responder. Outcomes including mortality were not significantly different between responders and non-responders. Digital ischemia (1/21) and mesenteric ischemia (1/21) were observed.

**Conclusions:** Vasopressin loading may predict responses to its continuous administration in septic shock patients. Further investigations involving a safety analysis are needed.

## Introduction

Vasopressin is one of the strongest vasopressor agents used to treat septic shock (1). Its effects have been demonstrated in several randomized control trials (2–4). Surviving Sepsis Campaign guidelines recommend the use of vasopressin as a second- or third-line vasopressor after noradrenaline for septic shock (5). As the adverse effects of vasopressin, ischemia events, such as digital ischemia (6, 7), mesenteric ischemia (8, 9), myocardial ischemia, and alterations in circulation dynamics (10) may be induced by strong vasoconstriction. However, recent clinical studies suggested that these adverse events are less frequent than previously reported, except for digital ischemia, when the appropriate dosage is administered (11, 12).

Regarding the dosage and administration of vasopressin, recent guidelines recommend continuous infusion up to 0.03 U/min (1.8 U/h) (5) because adverse events were occasionally reported with the administration of a high dose of vasopressin (13). However, since the half-life of vasopressin is 10–35 min (14) and a minimum blood concentration is needed for vasoconstriction (15), more time is needed to reach a steady state and achieve an adequate increase in blood pressure than that by catecholamines when continuously administered. Therefore, vasopressin loading with a bolus administration is occasionally performed in emergency medicine when immediate increases in blood pressure are needed to maintain the circulation. In our facility, we administer a 1 U bolus of vasopressin followed by continuous administration to patients with septic shock in whom blood pressure is not maintained at the target with adequate noradrenaline.

The beneficial effects of vasopressin loading may not only be rapid increases in blood pressure. Since responses to the administration of vasopressin may differ among patients, some may show marked improvements (1), whereas others do not (16). By identifying responders and non-responders to vasopressin loading, it may be possible to predict responses to its continuous administration, i.e., continuous administration may be effective for responders, while other strategies may be needed for non-responders.

Therefore, we herein retrospectively analyzed 21 consecutive cases of septic shock for which >0.2 μg/kg/min of noradrenaline was needed and vasopressin was introduced with loading. We hypothesized that vasopressin loading may be effective for predicting responses to its continuous administration. Outcomes and adverse events with immediate responses in blood pressure after vasopressin loading were assessed to investigate its significance and safety.

## Materials and Methods

This was a single-center retrospective study of patients with septic shock to whom vasopressin was administered with bolus loading. Consecutive cases of septic shock (sepsis-3) in the Hitachi General Hospital Emergency and Critical Care Center between August and October 2020, for which >0.2 μg/kg/min noradrenaline was administered and vasopressin was introduced with bolus loading, were analyzed. Patients administered vasopressin without bolus loading were excluded. In our facility, the introduction of vasopressin was only considered for cases of septic shock for which minimum noradrenaline >0.2 μg/kg/min was administered and additional doses were expected to be needed. In our clinical practice, vasopressin was administered as a 1 U bolus for loading, followed by its continuous administration at 1 U/h.

We classified patients into responders and non-responders based on hemodynamic changes with vasopressin loading. We extracted data on blood pressure (systolic, diastolic, and mean on arterial line monitoring) and heart rate just before and 1 min after 1 U vasopressin loading. Blood pressure was monitored and recorded using a radial or femoral arterial line continuously in all cases. Furthermore, the catecholamine index (CAI) [dopamine + dobutamine + (noradrenaline + adrenaline) × 100 μg/kg/min] (17) at pre-loading and 2, 4, and 6 h after the introduction of vasopressin, urine output every 2 h after the introduction of vasopressin, mortality, and the lengths of intensive care unit (ICU) and hospital stays were analyzed for outcomes. The proportion of patients with ΔCAI <0 6 h after the initiation of vasopressin, i.e., catecholamine doses were reduced due to the administration of vasopressin, was the primary outcome. We set a mean arterial pressure (MAP) increase of 18 mmHg 1 min after vasopressin loading as the cut-off of responders/non-responders for exploration, such that there was no case with changes in ΔCAI <0 at 2, 4, or 6 h in any non-responders. Responders were defined as those with a MAP change ≥18 mmHg 1 min after vasopressin loading, and non-responders as those with MAP <18 mmHg after loading. Digital ischemia, mesenteric ischemia, and myocardial ischemia were observed systematically by ICU nurses in this study period. Digital ischemia was visually checked every 4 h when patients stayed in the ICU. Mesenteric ischemia, defined as obvious ischemia, was confirmed by examinations including computed tomography. Myocardial ischemia, defined as acute ST-segment elevations, was confirmed by a 12-lead electrocardiogram or elevated cardiac enzymes. After discharge from the ICU, patients were evaluated from a review of medical records during the entire admission period. Outcome data were compared between responders and non-responders.

Regarding patient baseline information, age, sex, height, weight, the infection focus, and the presence/absence of cardiac failure, coronary artery disease (CAD), peripheral artery occlusion disease (PAOD), and immune disease were extracted. The sequential organ failure assessment (SOFA) score (18), Acute Physiology and Chronic Health Disease Classification System (APACHEII) score (19), modified shock index (heart rate/MAP)(20), body temperature, lactate, C-reactive protein (CRP), white blood cell (WBC) counts, albumin, platelet counts, the prothrombin time international normalized ratio (PT-INR), sodium, potassium, chloride, and blood glucose on the day of vasopressin use were evaluated. Steroid use equivalent to >40 mg/day of prednisolone, mechanical ventilation, renal replacement therapy (RRT), and extracorporeal membrane oxygenation (ECMO) were assessed as adjunctive therapies. These baseline information were also compared between responders and non-responders.

Since this was exploratory research, the sample size was not calculated and clinical practice was performed as usual. The present study was approved by our hospital ethics board (2017–19). Patients were included for analysis using an opt-out form.

The significance of differences was evaluated using the Student's *t*-test and Fisher's exact test for parametric data. The Mann–Whitney U test was performed for non-parametric data. The normality of the distribution of each parameter was assessed using the Shapiro–Wilk test. A *post-hoc* power analysis was conducted for the primary outcome, ischemia events, and severity scores. All statistical analyses were conducted using software (JMP 14; SAS Institute Inc., Cary, NC, USA). Results were expressed as a mean ± standard deviation or median (interquartile range). *P*-values <0.05 were considered to be significant and indicated with ^*^.

## Results

**Figure 1:**
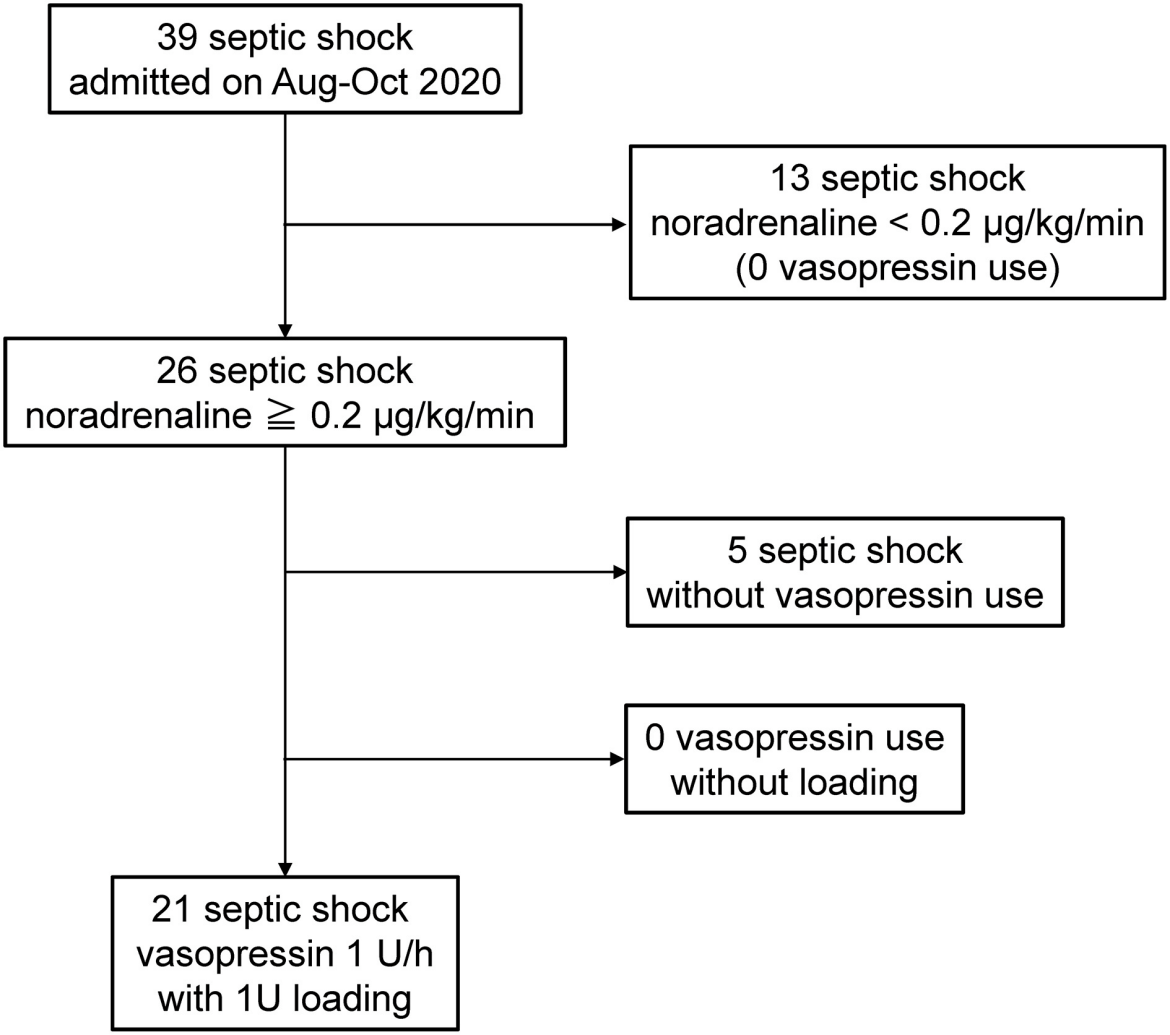
Study outline. Twenty-one consecutive patients with septic shock administered vasopressin with loading under noradrenaline >0.2 μg/kg/min. Bolus loading with 1 U following by continuous administration with 1 U/h was performed for all patients.

The patient extraction outline is shown in Figure 1. In the study period, 39 patients with septic shock defined as sepsis-3 were admitted to our Emergency and Critical Care Center. Twenty-six patients required noradrenaline >0.2 μg/kg/min, and 21 of these patients administered vasopressin were included in the present study. In this period, no patient was administered vasopressin without bolus loading. The noradrenaline dose before vasopressin administration was 0.35 ± 0.12 μg/kg/min. Bolus loading of 1 U following by the continuous administration of 1 U/h was performed for all patients administered vasopressin. The baseline characteristics of the 21 patients are shown in Supplementary Table 1. Hemodynamic changes before/after vasopressin loading and outcomes were shown in Supplementary Table 2. No obvious circulatory alteration was observed in any cases with 1 U vasopressin loading; however, MAP increased to higher than 120 mmHg after loading in two cases (Supplementary Table 2), and subsequently decreased to <100 mmHg within the next few minutes.

Based on the definition of a responder to vasopressin loading of a MAP change ≥18 mmHg from before to 1 min after loading, 14 responders (66.7%) and 7 non-responders (33.3%) were identified. Differences in baseline characteristics between responders and non-responders are shown in Table 1. No significant difference was observed in age. A male predominance, larger body size, severity and pneumonia as the infection focus were observed in non-responders. Power (1−β) for SOFA and APACHEII were 0.257 and 0.055, respectively. No significant differences were noted in the noradrenaline dose on vasopressin administration, cardiac failure, CAD, PAOD, or immunodeficiency. However, the time between vasopressin administration and shock onset was significantly longer in responders.

**Table 1:** Differences in baseline characteristics between vasopressin loading responders and non-responders.

***n***	**Responder**	**Non-Responder**	***p-*value**
	**14**	**7**	
age	78.7 ± 7.6	78.1 ± 9.8	0.88
male	5 (35.7%)	5 (71.4%)	0.18
height (cm)	156.2 ± 10.9	160.5 ± 11.2	0.4
weight (kg)	52.5 (46.9, 57)	58 (41.1, 78.8)	0.85
SOFA	7 (5.8, 8.5)	11 (4, 14)	0.22
APACHEII	17.5 (13.5, 24)	21 (14, 28)	0.41
modified shock index	1.45 (1.11, 1.72)	1.58 (1.34, 2.49)	0.28
noradrenaline dose on vasopressin administration (μg/kg/min)	0.34 ± 0.15	0.37 ± 0.08	0.63
time of vasopressin administration from shock onset (hours)	6.5 (3, 10)	2 (2, 2.5)	0.017^*^
immunodeficiency	3 (21.4%)	1 (14.3%)	1
Infection focus			0.5
pneumonia	4 (28.6%)	5 (71.4%)	
urinary tract infection	3 (21.4%)	1 (14.3%)	
CRBSI	1 (7.1%)	0 (0%)	
cholangitis	1 (7.1%)	1 (14.3%)	
peritonitis	1 (7.1%)	0 (0%)	
meningitis	1 (7.1%)	0 (0%)	
unknown	3 (21.4%)	0 (0%)	
cardiac failure	4 (28.6%)	2 (28.6%)	1
CAD	1 (7.1%)	1 (14.3%)	1
PAOD	0 (0%)	1 (7.1%)	1
mechanical ventilation	10 (71.4%)	5 (71.4%)	1
RRT	3 (21.4%)	2 (28.6%)	1
ECMO	0 (0%)	1 (14.3%)	0.33
body temperature (°C)	37.8 ± 1.1	38.5 ± 1.7	0.26
lactate (mmol/l)	3.0 (1.6, 4.2)	3.9 (2.3, 10.4)	0.29
glucose (mg/dl)	136 (98, 175)	221 (190, 258)	0.0032^*^
PT-INR	1.4 ± 0.4	2.0 ± 0.8	0.040^*^

*SOFA, sequential organ failure assessment; APACHE, acute physiology and chronic health evaluation; CRBSI, catheter-related bloodstream infection; CAD, coronary artery disease; PAOD, peripheral arterial obstructive disease; RRT, renal replacement therapy; ECMO, extracorporeal membrane oxygenation; PT-INR, prothrombin time international normalized ratio*.

* *p < 0.05*.

**Figure 2:**
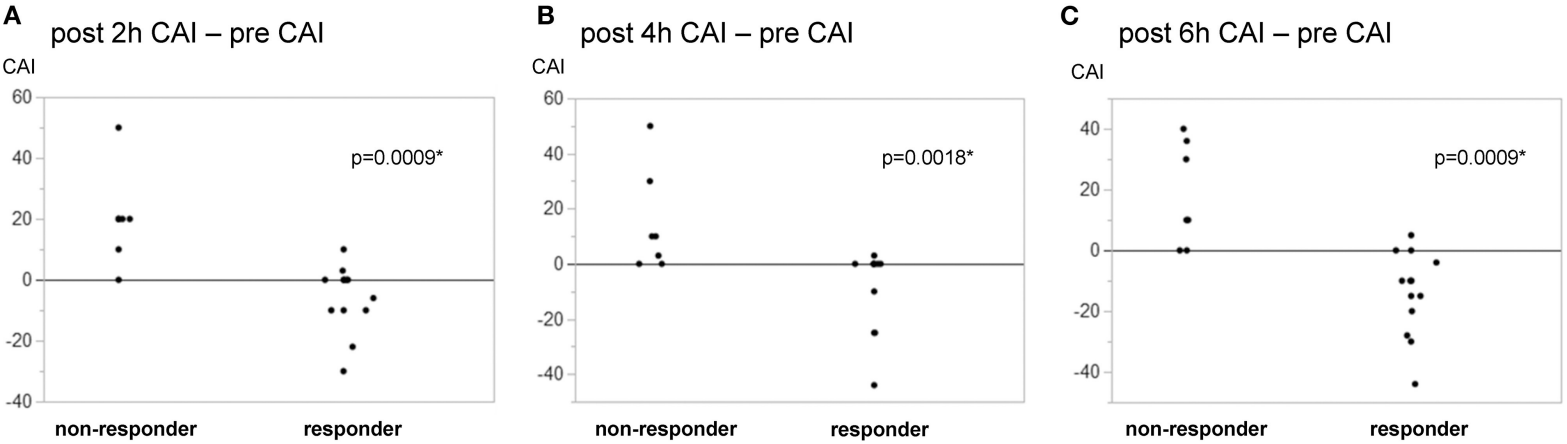
Changes in the catecholamine index in responders/non-responders to vasopressin loading. Responders to vasopressin loading were defined by a MAP change ≥18 mmHg from before to 1 min after loading. Fourteen responders (66.7%) and 7 non-responders were identified. A change was observed in the catecholamine index (CAI) [dopamine + dobutamine + (noradrenaline + adrenaline) × 100 μg/kg/min] from pre-loading to 2, 4, and 6 h after the initiation of vasopressin. **(A)** post 2-h—pre. **(B)** post 4-h—pre. **(C)** post–pre. Changes in CAI significantly differed between responders and non-responders at 2, 4, and 6 h. *p < 0.05.

Table 2 shows the outcomes of vasopressin loading and continuous administration. The vasopressin dose (1 U) normalized by body weight was similar in responders and non-responders. The duration of the vasopressin infusion did not significantly differ between responders and non-responders, and vasopressin was administered to all patients for 6 h. The primary outcome of the proportion of patients with ΔCAI <0 6 h after the initiation of vasopressin accounted for 71.4% of responders and 0% of non-responders, with a significant difference (*p* = 0.0039). In the *post-hoc* analysis, power (1−β) for the primary outcome was 0.999. The proportion of patients with ΔCAI <0 at 2 and 4 h accounted 42.9 and 50.0% of responders, respectively, and 0% of non-responders at both time points. ΔCAI are shown in Figure 2. Median ΔCAI changes 2, 4, and 6 h after the administration of vasopressin were 0, −5, and −10 in responders and +20, +10, and +10 in non-responders, respectively, with significant differences (Figure 2). Responses to vasopressin loading correlated with ΔCAI after the initiation of its administration.

**Table 2:** Outcome differences between vasopressin loading responders and non-responders.

***n***	**Responder**	**Non-Responder**	***p*-value**
	**14**	**7**	
**Primary outcome**			
post 6 h CAI—pre CAI <0	10 (71.4%)	0 (0%)	0.0039^*^
pre SBP (mmHg)	85.6 ± 17.3	84.8 ± 18.1	0.93
pre DBP (mmHg)	45.4 ± 11.7	44.1 ± 6.5	0.79
pre MAP (mmHg)	59.9 ± 13.1	56.7 ± 9.2	0.58
pre HR (beats/min)	87.7 ± 19.3	101.7 ± 20.3	0.14
CVP (mmHg)	8.5 (4, 13.5)	11 (3.5, 15.3)	0.67
pre CAI	34.6 ± 15.2	37.1 ± 7.6	0.32
post 2 h CAI—pre CAI <0	6 (42.9%)	0 (0%)	0.061
post 4 h CAI—pre CAI <0	7 (50%)	0 (0%)	0.047^*^
vasopressin (U/kg)	0.019 ± 0.004	0.019 ± 0.006	0.99
vasopressin (hours)	44 (27.3, 84)	38 (26, 56)	0.65
mortality	6 (42.9%)	3 (42.9%)	1
(DNAR after treatment)	6 (42.9%)	2 (28.6%)	0.66
ICU stay (days)	8 (5.8, 11.8)	7 (4, 7)	0.29
hospital stay (days)	23 (13.3, 37.8)	11 (4, 15)	0.13
digital ischemia	0 (0%)	1 (14.3%)	0.33
mesenteric ischemia	1 (7.1%)	0 (0%)	1
myocardial ischemia	0 (0%)	0 (0%)	1
urine output pre-2 h	85 (16, 143)	50 (20, 100)	0.88
urine output 2–4 h	73 (7.5, 158)	50 (5, 250)	0.94

*CAI, catecholamine index; SBP, systolic blood pressure, DBP, diastolic blood pressure; MAP, mean arterial pressure; HR, heart rate; CVP, central venous pressure; DNAR, Do Not Attempt Resuscitation; ICU, intensive care unit*.

* *p <0.05*.

No significant differences were observed in other outcomes, including mortality (Table 2). Digital ischemia, mesenteric ischemia, and myocardial ischemia as adverse events did not significantly differ between responders and non-responders. Power (1−β) for all ischemia events was 0.246. In all patients in the present study for whom vasopressin loading was performed, few adverse events were observed. Mesenteric ischemia was noted in patients with PAOD (Supplementary Tables 1, 2). Causality was unclear in all cases. Significant differences were also observed in blood glucose and PT-INR.

## Discussion

Based on the bolus loading of 1 U vasopressin in patients with septic shock for whom the administration of vasopressin was needed under noradrenaline >0.2 μg/kg/min, two-thirds of patients were identified as responders and one-third as non-responders, and bolus loading correlated with the need for another catecholamine dose after the administration of vasopressin. The primary outcome of the proportion of patients with ΔCAI <0 6 h after the initiation of vasopressin was significantly higher in responders. Ischemia events may not be excessive with vasopressin loading.

Rapid increases in blood pressure may be achieved with vasopressin loading. As shown in the change in blood pressure for 1 min after loading, blood pressure increased more rapidly with loading than with continuous administration without loading. An increase in blood pressure after continuous administration without loading may only be observed when a steady state is achieved with priming and the appropriate preparation of the infusion.

Vasoconstriction and blood pressure increases by vasopressin may only be achieved in humans when plasma vasopressin concentrations are higher than 50 pg/ml (15). Noradrenaline and other catecholamines may induce vasoconstriction linearly from the lowest concentration (21). These differences in the concentration-vasoconstriction relationship are caused by vasopressin and catecholamines receptors, namely, V1 and α1 (22). Therefore, the time needed for vasopressin to increase blood pressure before an adequate blood concentration is attained may be longer than that by noradrenaline. Moreover, the half-life of catecholamines, including noradrenaline, is 2 min (23), while that of vasopressin is 10–35 min (14); therefore, loading appears to be appropriate for the early achievement of the blood pressure target. The bolus administration of vasopressin has not yet been examined in detail. Terlipressin, an analogue of vasopressin (24) with a longer half-life of 50 min (25), has been administered with bolus loading in clinical trials (26) and animal studies (27). Although the risk of ischemia increases at high doses of terlipressin (28), it may be safe when administered at an appropriate dose. The bolus administration of vasopressors has been recommended in emergency and critical care cases (29).

However, vasopressin loading is not discussed in the Surviving Sepsis Campaign guidelines (5) due to the adverse effects associated with its administration at a high dose. High-dose vasopressin may induce digital (6, 7), mesenteric (8, 9), and myocardial ischemia (10). Previous case series and clinical trials suggested that the administration of more than 0.05 U/min increased the risk of these adverse effects (11), and, thus, <0.03 U/min is recommended (5). However, few studies have investigated the effects of a vasopressin bolus. To the best of our knowledge, there has only been one study on 7 cases for which a bolus of 50 mU/kg vasopressin was administered (30); 4 out of 7 cases died due to mesenteric ischemia. In contrast, the mortality rate in the present study was 40% with a lower bolus dose of 1 U because patients required noradrenaline >0.2 μg/kg/min and another vasopressor. The rates of digital and mesenteric ischemia were similar to that reported by VASST (3). However, since this was a preliminary analysis of a limited case series, the safety of vasopressin loading needs to be investigated in a larger number of patients. Furthermore, in two out of 21 cases in the present study, MAP increased to higher than 120 mmHg after loading. A transient increase in noradrenaline may be associated with a delayed blood pressure increase and vasopressin may cause excessive vasoconstriction. This condition may decrease cardiac output and oxygen delivery; therefore, it needs to be considered prior to vasopressin loading.

In the present study, two-thirds of patients with septic shock for whom noradrenaline >0.2 μg/kg/min and another vasopressor were required responded to vasopressin loading. In all responders, another catecholamine was not needed after the administration of vasopressin. On the other hand, it was not possible to reduce the dose of catecholamines in the remaining one-third of non-responders, the majority of whom still required additional catecholamine doses even after the administration of vasopressin. Differences in responses to the administration of vasopressin have been attributed to the depletion of vasopressin and a cortisol insufficiency (16). Prognostic differences in the use of vasopressin for septic shock were previously attributed to hormonal differences in clinical trials (3, 4). We did not assess blood antidiuretic hormone (ADH) or cortisol levels or perform an adrenocorticotropic hormone (ACTH) loading test because this was a retrospective study. However, the time between vasopressin administration and shock onset was significantly longer in responders in the present study. One reason for this may be the depletion of vasopressin after the onset of septic shock. Furthermore, irrespective of hormonal changes, it may be necessary to monitor continuous vasopressin administration in responders and immediately introduce another procedure to increase blood pressure (such as adrenaline or a circulatory assist device) in non-responders, suggesting the usefulness of vasopressin loading to predict responses to vasopressin. Since another potential advantage of vasopressin is the prevention of tachyarrhythmia by reducing the requirement for noradrenaline (12), vasopressin responder predictions may contribute to decreases in the administration of unnecessary noradrenaline and tachycardia.

There are several limitations that need to be addressed. This was a retrospective analysis of a case series in a limited time period. The sample size was small and underpowered. Therefore, a prospective study that examines the safety of vasopressin loading and its effects on patient prognosis is needed, and we are now preparing the VAsopressin LOading for Refractory septic shock VALOR trial. It will be important to assess blood ADH and cortisol levels and perform an ACTH loading test before vasopressin loading and compare the data obtained between responders and non-responders. Furthermore, vasopressin was administered at a dose of 1 U for loading and 1 U/h for continuous infusion. Since Japanese ICU patients are often smaller and older than those in Western countries, a dose of 0.03 U/min (1.8 U/h) may be too high; therefore, we adopted the described protocol. The effects of vasopressin may markedly change depending on the dosage administered (31, 32), and, hence, vasopressin loading at other dosages needs to be investigated. In addition, several factors, including norepinephrine and vasopressin doses, may influence responses to vasopressin and other outcomes (33).

## Conclusions

Vasopressin loading may predict responses to its continuous administration in septic shock patients. Further investigations involving a safety analysis are needed.

## Data Availability Statement

The original contributions presented in the study are included in the article/Supplementary Material, further inquiries can be directed to the corresponding author/s.

## Ethics Statement

The studies involving human participants were reviewed and approved by Hitachi General Hospital. Written informed consent for participation was not required for this study in accordance with the national legislation and the institutional requirements.

## Author Contributions

KN: conception of the study, interpretation, and drafting of the manuscript. HNak, HNar, MM, YT, TS, and HH: performance of clinical practices. TA, MH, and KY: data analysis and supervision of the study. All authors: have read and approved the manuscript.

## Conflict of Interest

HN and TS were employed by TXP Medical Co., Ltd. The remaining authors declare that the research was conducted in the absence of any commercial or financial relationships that could be construed as a potential conflict of interest.
