# Supplementary material for: Vasopressin Loading for Refractory Septic Shock: A Preliminary Analysis of a Case Series
Source: Front Med (Lausanne). 2021 May 4;8:644195. doi: 10.3389/fmed.2021.644195 (PMC8129171; doi:10.3389/fmed.2021.644195)
Supplement: Supplementary file 1 [file Table_1.docx]

**Table 1. Basic characteristics of 21 patients with vasopressin loading**

SOFA, sequential organ failure assessment; APACHE, acute physiology and chronic health evaluation; CRP, C reactive protein; WBC, white blood cell; PT-INR, prothrombin time international normalized ratio; RRT, renal replacement therapy; ECMO, extracorporeal membrane oxygenation; UTI, urinary tract infection; CRBSI, catheter-related bloodstream infection; CAD, coronary artery disease; PAOD, peripheral arterial obstructive disease

| **case** | **age** | **sex** | **height cm** | **weight kg** | **infection focus** | **cardiac failure** | **CAD** | **PAOD** | **immune disease** | **SOFA** | **APACHEII** | **body temperature °C** | **lactate mmol/l** | **CRP mg/dl** | **WBC /μl** | **Alb mg/dl** | **Plt ×10^4^/μl** | **PT-INR** | **Na mmol/l** | **K mmol/l** | **Cl mmol/l** | **Glucose mg/dl** | **steroid use** | **mechanical ventilation** | **ventilator days** | **RRT** | **RRT days** | **ECMO** |
| --- | --- | --- | --- | --- | --- | --- | --- | --- | --- | --- | --- | --- | --- | --- | --- | --- | --- | --- | --- | --- | --- | --- | --- | --- | --- | --- | --- | --- |
| 1 | 79 | f | 155 | 53 | UTI | + | - | - | lymphoma | 4 | 25 | 36.3 | 11.5 | 6.91 | 5800 | 2.8 | 2.9 | 2.4 | 123 | 5.1 | 94 | 91 | − | + | 1 | − |  | − |
| 2 | 77 | m | 169 | 86 | UTI | − | - | - |  | 15 | 32 | 37.9 | 4 | 4.2 | 16400 | 1.7 | 11.3 | 1.7 | 140 | 4.6 | 105 | 99 | − | + | 1 | + |  | − |
| 3 | 69 | m | 171 | 47 | unknown | − | - | - |  | 6 | 5 | 39.3 | 4 | 1.32 | 1340 | 2.6 | 33.5 | 1 | 138 | 4.4 | 107 | 162 | − | − |  | − |  | − |
| 4 | 89 | f | 142 | 45 | pneumonia | − | - | - |  | 2 | 14 | 41.8 | 4.4 | 0.27 | 14400 | 2.6 | 16.5 | 1.3 | 145 | 3.6 | 100 | 135 | − | + | 6 | − |  | − |
| 5 | 82 | f | 160 | 52 | infectious endocarditis | + | - | - |  | 7 | 18 | 37.6 | 1.1 | 10.89 | 8300 | 1.7 | 14 | 1.3 | 136 | 3.6 | 105 | 137 | − | + | 22 | + | 30 | − |
| 6 | 76 | m | 158 | 41.1 | UTI | − | - | - |  | 14 | 28 | 36.7 | 10.4 | 2.37 | 11500 | 3.3 | 13.1 | 2 | 132 | 4.7 | 101 | 242 | − | + | 4 | + | 3 | − |
| 7 | 67 | m | 163 | 58 | cholangitis | − | - | - |  | 9 | 32 | 37.7 | 3.9 | 18.33 | 26500 | 2.9 | 8 | 1.7 | 136 | 3.2 | 108 | 196 | − | − |  | − | 0 | − |
| 8 | 75 | m | 169 | 78.8 | pneumonia | + | + | - |  | 11 | 21 | 39.2 | 2.2 | 3.01 | 11100 | 3 | 15.4 | 2.1 | 139 | 4.2 | 111 | 190 | − | + | 3 | − |  | − |
| 9 | 81 | f | 145 | 40.1 | cholangitis | − | - | - |  | 10 | 14 | 38.5 | 2.6 | 13.14 | 22600 | 2.6 | 9.2 | 1.8 | 139 | 3.1 | 108 | 172 | − | − |  | − |  | − |
| 10 | 80 | m | 165 | 60 | pneumonia | − | - | - |  | 6 | 24 | 38.7 | 2.2 | 0.67 | 11800 | 3.1 | 22.5 | 1.4 | 136 | 5.5 | 104 | 134 | − | + | 15 | − |  | − |
| 11 | 93 | f | 153 | 35 | pneumonia | + | - | - |  | 11 | 25 | 37.9 | 13.9 | 3.4 | 10600 | 2.1 | 21.2 | 2 | 143 | 3.3 | 108 | 221 | − | + | 2 | − |  | − |
| 12 | 85 | f | 140 | 50 | unknown | − | - | - |  | 8 | 24 | 36.1 | 3.2 | 14.71 | 27400 | 3.1 | 4.2 | 1.33 | 148 | 3.4 | 116 | 220 | − | + | 14 | − | 0 | − |
| 13 | 91 | f | 162 | 56 | pneumonia | + | - | - |  | 6 | 17 | 37.8 | 1 | 15.08 | 12000 | 3.6 | 15.9 | 1 | 137 | 4.9 | 105 | 188 | − | + | 6 | − |  | − |
| 14 | 83 | f | 150 | 46.5 | meningitis | − | - | - | rheumatoid arthritis | 5 | 17 | 37.9 | 0.7 | 20.69 | 10700 | 2.6 | 17.2 | 1.2 | 129 | 3.9 | 98 | 89 | − | − |  | − |  | − |
| 15 | 85 | f | 142 | 53 | UTI | − | + | - |  | 2 | 7 | 39.1 | 5.9 | 6.52 | 10700 | 2.6 | 8.6 | 1.18 | 138 | 2.8 | 102 | 126 | − | − |  | − |  | − |
| 16 | 62 | f | 147 | 40 | unknown | − | - | + |  | 10 | 15 | 36.3 | 1.7 | 13.73 | 6600 | 2.7 | 10.1 | 1.18 | 139 | 2.8 | 106 | 142 | + | + | 5 | − |  | − |
| 17 | 81 | f | 152 | 50 | CRBSI | + | - | - |  | 7 | 20 | 37.1 | 4.6 | 26.14 | 18000 | 2.6 | 6.1 | 1.54 | 139 | 4 | 106 | 107 | − | + | 5 | + | 5 | − |
| 18 | 79 | m | 162 | 59 | pneumonia | − | - | - | cirrhosis | 16 | 20 | 37.6 | 3.9 | 22.25 | 23200 | 2.4 | 0.5 | 1 | 136 | 4.2 | 109 | 305 | − | + | 13 | + | 7 | + |
| 19 | 78 | m | 155 | 56 | pneumonia | − | - | - | pachymeningitis cervicalis | 7 | 12 | 38 | 4 | 2.18 | 3800 | 2.8 | 11.4 | 1 | 138 | 4.1 | 110 | 96 | − | + | 4 | − |  | − |
| 20 | 68 | m | 177 | 90 | pneumonia | − | - | - |  | 4 | 12 | 38.9 | 2.3 | 20.87 | 7300 | 2.7 | 10.2 | 3.6 | 127 | 3.8 | 97 | 258 | − | − |  | − |  | − |
| 21 | 69 | m | 174 | 83.1 | peritonitis | − | - | - |  | 7 | 21 | 39.2 | 2.88 | 7.2 | 16500 | 2.5 | 26.6 | 1.32 | 134 | 3.9 | 105 | 182 | − | + | 5 | − |  | − |
