# Supplementary material for: Vasopressin Loading for Refractory Septic Shock: A Preliminary Analysis of a Case Series
Source: Front Med (Lausanne). 2021 May 4;8:644195. doi: 10.3389/fmed.2021.644195 (PMC8129171; doi:10.3389/fmed.2021.644195)
Supplement: Supplementary file 2 [file Table_2.docx]

**Table 2 Outcomes of 21 patients with vasopressin loading**

SBP, systolic blood pressure, DBP, diastolic blood pressure; MAP, mean arterial pressure; HR, heart rate; CVP, central venous pressure; CAI, catecholamine index; ICU, intensive care unit

| **case** | **vasopressin hours** | **vasopressin from shock onset**  **hours** | **pre SBP mmHg** | **pre DBP mmHg** | **pre MAP mmHg** | **pre HR /min** | **CVP mmHg** | **post SBP mmHg** | **post DBP mmHg** | **post MAP mmHg** | **post HR /min** | **pre NOA**  **μg/kg/min** | **pre CAI** | **post 2h CAI** | **post 4h CAI** | **post 6h CAI** | **urine output pre-2h** | **urine output 2h-4h** | **urine output 4h-6h** | **digital ischemia** | **mesenteric ischemia** | **myocardial ischemia** | **mortality** | **ICU stay days** | **hospital stay days** |
| --- | --- | --- | --- | --- | --- | --- | --- | --- | --- | --- | --- | --- | --- | --- | --- | --- | --- | --- | --- | --- | --- | --- | --- | --- | --- |
| 1 | 29 | 4.0 | 80 | 42 | 60 | 76 |  | 184 | 96 | 120 | 65 | 0.2 | 20 | 20 | 20 | 20 | 150 | 150 | 150 | − | − | − | dead | 4 | 4 |
| 2 | 57 | 15.0 | 53 | 26 | 36 | 115 |  | 136 | 63 | 85 | 129 | 0.5 | 60 | 60 | 60 | 60 | 2 | 0 | 3 | − | − | − | dead | 3 | 20 |
| 3 | 45 | 10.0 | 88 | 55 | 65 | 88 | 1 | 142 | 81 | 104 | 73 | 0.3 | 30 | 20 | 20 | 20 | 100 | 80 | 80 | − | − | − | survived | 6 | 50 |
| 4 | 38 | 2.0 | 88 | 36 | 49 | 99 | 2 | 126 | 46 | 65 | 99 | 0.6 | 40 | 60 | 40 | 40 | 100 | 250 | 270 | − | − | − | survived | 6 | 68 |
| 5 | 108 | 168.0 | 86 | 47 | 66 | 124 | 8 | 108 | 64 | 84 | 118 | 0.3 | 30 | 20 | 20 | 15 | 20 | 10 | 10 | − | − | − | dead | 22 | 32 |
| 6 | 45 | 20.0 | 109 | 44 | 57 | 86 | 15 | 124 | 52 | 67 | 89 | 0.3 | 30 | 50 | 40 | 40 | 0 | 0 | 2 | − | − | − | dead | 4 | 4 |
| 7 | 67 | 2.0 | 106 | 53 | 73 | 98 | 4 | 127 | 64 | 86 | 77 | 0.4 | 40 | 50 | 50 | 50 | 250 | 150 | 150 | − | − | − | survived | 7 | 15 |
| 8 | 56 | 2.0 | 60 | 38 | 46 | 130 |  | 87 | 49 | 59 | 128 | 0.5 | 30 | 80 | 80 | 60 | 30 | 5 | 20 | − | − | − | survived | 7 | 11 |
| 9 | 34 | 10.0 | 97 | 43 | 62 | 112 | 7 | 154 | 73 | 103 | 110 | 0.2 | 20 | 20 | 10 | 5 | 150 | 180 | 100 | − | − | − | survived | 6 | 35 |
| 10 | 12 | 8.0 | 109 | 72 | 88 | 89 | 14 | 145 | 89 | 110 | 88 | 0.5 | 50 | 20 | 6 | 6 | 200 | 270 | 250 | − | − | − | survived | 17 | 46 |
| 11 | 26 | 2.5 | 68 | 40 | 51 | 127 | 14 | 74 | 44 | 55 | 117 | 0.5 | 30 | 50 | 33 | 66 | 20 | 20 | 10 | + | − | − | dead | 2 | 2 |
| 12 | 96 | 3.0 | 98 | 50 | 70 | 77 | 10 | 216 | 116 | 158 | 77 | 0.3 | 30 | 8 | 5 | 0 | 100 | 100 | 200 | − | − | − | dead | 14 | 14 |
| 13 | 120 | 6.0 | 75 | 44 | 57 | 57 | 16 | 102 | 57 | 75 | 61 | 0.3 | 30 | 30 | 30 | 30 | 140 | 250 | 60 | − | − | − | dead | 11 | 11 |
| 14 | 34 | 5.0 | 105 | 42 | 57 | 88 | 16 | 146 | 60 | 90 | 83 | 0.2 | 20 | 20 | 20 | 16 | 80 | 100 | 100 | − | − | − | survived | 5 | 26 |
| 15 | 11 | 2.0 | 87 | 36 | 52 | 88 | 12 | 126 | 58 | 80 | 88 | 0.4 | 30 | 40 | 30 | 10 | 35 | 65 | 80 | − | − | − | survived | 7 | 14 |
| 16 | 80 | 3.0 | 70 | 40 | 50 | 83 | -1 | 102 | 63 | 76 | 93 | 0.5 | 50 | 53 | 53 | 55 | 0 | 0 | 0 | − | + | − | dead | 10 | 6 |
| 17 | 43 | 2.5 | 55 | 34 | 43 | 57 | 3 | 102 | 69 | 80 | 70 | 0.6 | 65 | 55 | 40 | 37 | 0 | 0 | 100 | − | − | − | survived | 11 | 31 |
| 18 | 26 | 2.0 | 81 | 51 | 62 | 98 | 8 | 90 | 54 | 68 | 98 | 0.5 | 50 | 70 | 80 | 90 | 100 | 260 | 200 | − | − | − | dead | 12 | 13 |
| 19 | 79 | 7.0 | 95 | 63 | 75 | 83 | 7 | 132 | 88 | 104 | 98 | 0.3 | 30 | 30 | 30 | 20 | 90 | 60 | 60 | − | − | − | survived | 7 | 14 |
| 20 | 36 | 2.0 | 82 | 47 | 59 | 74 | 16 | 103 | 58 | 72 | 79 | 0.4 | 40 | 40 | 40 | 40 | 50 | 50 | 50 | − | − | − | survived | 7 | 7 |
| 21 | 22 | 7.0 | 100 | 42 | 57 | 91 | 9 | 165 | 64 | 92 | 75 | 0.2 | 20 | 14 | 10 | 10 | 42 | 14 | 25 | − | − | − | survived | 9 | 51 |
